# Evaluation of an aortic valve prosthesis: Fluid-structure interaction or structural simulation?

**DOI:** 10.1016/j.jbiomech.2017.04.004

**Published:** 2017-06-14

**Authors:** Giulia Luraghi, Wei Wu, Francesco De Gaetano, Josè Felix Rodriguez Matas, Geoff D. Moggridge, Marta Serrani, Joanna Stasiak, Maria Laura Costantino, Francesco Migliavacca

**Affiliations:** a Laboratory of Biological Structure Mechanics (LaBS), Department of Chemistry, Materials and Chemical Engineering “Giulio Natta”, Politecnico di Milano, Milan, Italy; b Department of Chemical Engineering and Biotechnology, University of Cambridge, Cambridge, UK

**Keywords:** Fluid-structure interaction, Polymeric heart valve, Finite element analysis, Cardiovascular mechanics

## Abstract

Bio-inspired polymeric heart valves (PHVs) are excellent candidates to mimic the structural and the fluid dynamic features of the native valve. PHVs can be implanted as prosthetic alternative to currently clinically used mechanical and biological valves or as potential candidate for a minimally invasive treatment, like the transcatheter aortic valve implantation. Nevertheless, PHVs are not currently used for clinical applications due to their lack of reliability. In order to investigate the main features of this new class of prostheses, pulsatile tests in an in-house pulse duplicator were carried out and reproduced *in silico* with both structural Finite-Element (FE) and Fluid-Structure interaction (FSI) analyses. Valve kinematics and geometric orifice area (GOA) were evaluated to compare the *in vitro* and the *in silico* tests. Numerical results showed better similarity with experiments for the FSI than for the FE simulations. The maximum difference between experimental and FSI GOA at maximum opening time was only 5%, as compared to the 46.5% between experimental and structural FE GOA. The stress distribution on the valve leaflets clearly reflected the difference in valve kinematics. Higher stress values were found in the FSI simulations with respect to those obtained in the FE simulation. This study demonstrates that FSI simulations are more appropriate than FE simulations to describe the actual behaviour of PHVs as they can replicate the valve-fluid interaction while providing realistic fluid dynamic results.

## 1 Introduction

In the arena of heart valve prostheses, bio-inspired polymeric heart valves (PHVs) are excellent candidates to mimic not only the shape, but also the structural and fluid dynamic behaviour of the native valve (Kuan et al., 2011). Indeed, they aim at combining the main advantages from the mechanical and biological valve prostheses. PHVs exhibit good fluid dynamics and hemocompatibility performances, the same as biological valves. PHVs are also potential candidate for transcatheter aortic valve replacement (TAVR) (Yousefi et al., 2016b), a minimally invasive treatment for patients with significant contraindications for standard surgery (Smith et al., 2011). TAVR, which is a proven technology nowadays, consists in the insertion of a stented valve in the aortic root using a catheter (Cribier et al., 2002). In both applications of PHVs, used as a traditional valve prosthesis or as a TAVR, a number of critical aspects influencing prosthesis performance are still present; they require further investigation. As a matter of fact, in spite of their promising ability to replicate the function of native valves (Ghanbari et al., 2009, Rahmani et al., 2012), PHVs are not currently used for clinical applications due to their lack of reliability (Kheradvar et al., 2015).

A thorough characterisation of the hydrodynamic behaviour of polymeric valves is required to understand the characteristics of the device, since the behaviour of a heart valve is influenced not only by the geometry of the leaflets and their material properties, but also by the fluid passing through the valve. In this regard, fluid-structure interaction (FSI) models are becoming increasingly important for biomedical engineering applications, in particular to study the dynamics of human heart valves (De Hart et al., 2003b). For these reasons, in this work we develop a computational FSI model of a PHV and compare the results with structural finite element (FE) simulations where the presence of the fluid is not considered. In the literature, a number of computational studies on prosthetic heart valves have been performed neglecting the blood flow across the prosthetic valve but simply considering hydrostatic pressures acting on the structure domain (Gunning et al., 2014, Wang et al., 2015, Morganti et al., 2014). At the same time, the number of studies considering fluid-structure interaction is increasing, for instance, studies on the behaviour of the aortic root in the presence of native valves (De Hart et al., 2003a, Marom et al., 2012, Ranga et al., 2006, Sturla et al., 2013, Weinberg and Kaazempur Mofrad, 2007), and a few on prosthetic valves (Bavo et al., 2016, Wu et al., 2016, Borazjani, 2013). However, with exception of the work by Wu et al., none of the previous works has included experimental validations.

The aim of this study is to demonstrate how the FSI methodology is more reliable than the stand-alone structural analysis to replicate *in vitro* tests of a polymeric aortic valve. In particular, (a) we conducted pulsatile tests in an *in-house* pulse duplicator built up based on the guidelines of the ISO5840:2015 Standard, (b) we reproduced the *in vitro* conditions with both structure and FSI simulations, in order to (c) compare the numerical results against experiments in terms of valve kinematics, while providing additional information such as stress distribution and velocity fields.

## 2 Material and methods

### 2.1 PHV valve

**Fig. 1:**
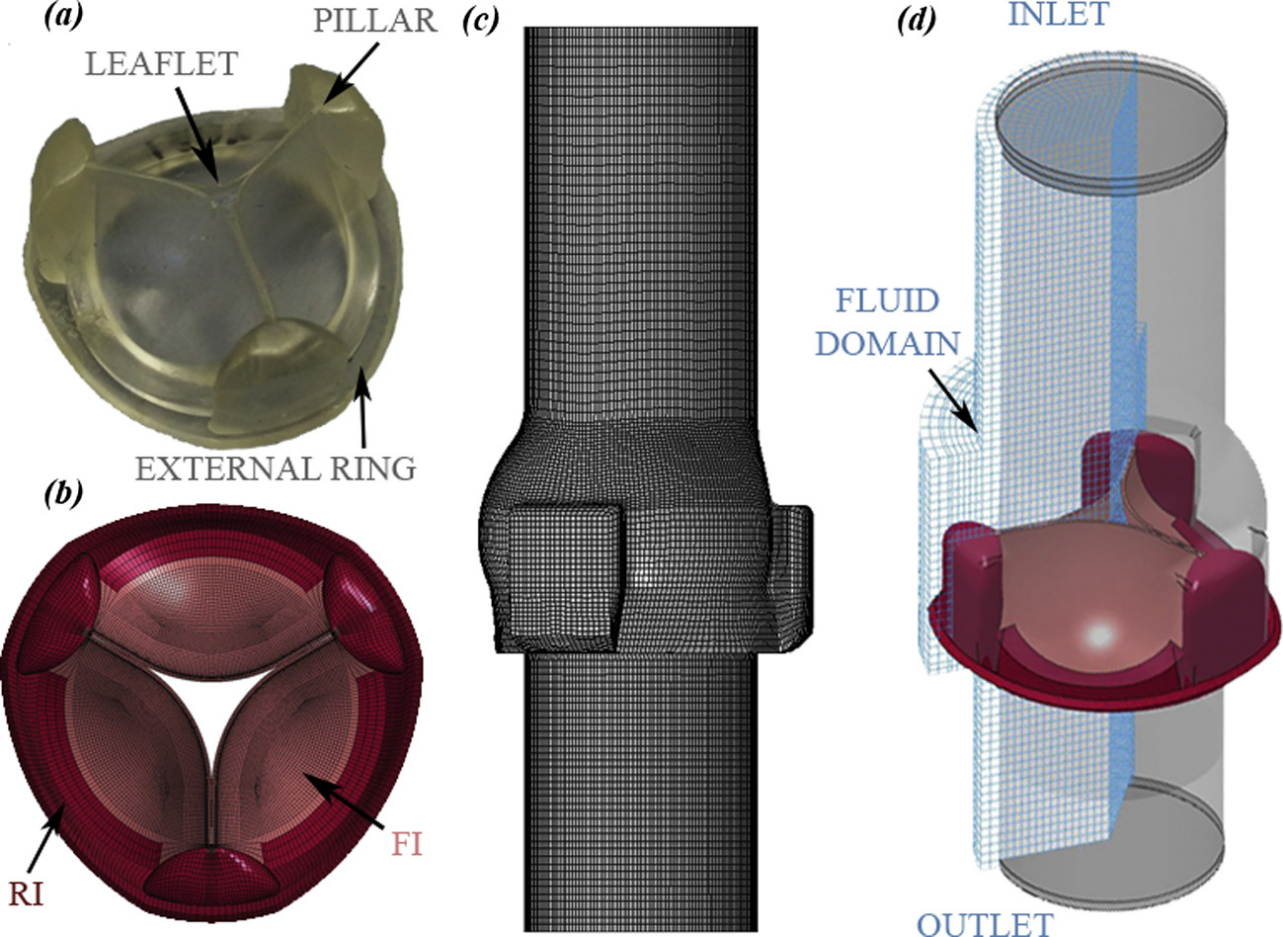
Prosthesis Heart Valve (PHV) sample with pillars, external ring and leaflets (*a*) and the corresponding (*b*) model of the PHV with reduced (RI) and fully integrated (FI) elements; mesh of the aortic valve housing (*c*); FSI model including the valve, the compartment and the fluid domain with inlet and outlet parts (*d*).

The PHV prototypes similar to those presented by De Gaetano et al. (2015a) made of styrenic block copolymer (SBP) have been considered in this work (Fig.1a). The PHVs are manufactured by moulding poly (styrene – ethylene – propylene – styrene) (SEPS) block copolymers with 22% percentage by mass (wt) polystyrene fraction. PHVs have extremely thin leaflets, which should hamper the flow as little as possible when opened, but need to prevent blood back-flow if closed. During closure, the leaflets are in mutual contact and a large transvalvular pressure gradient occurs. The leaflets are directly connected to the valve structure by three pillars as detailed in Fig.1a. For the valve taken into consideration in this work, the three leaflets have different average thickness (two with a thickness of 0.36 mm and one of 0.39 mm). This difference was due to imprecision during the fabrication process and was taken into account when creating the computational model of the valve.

### 2.2 Hydrodynamic tests

**Fig. 2:**
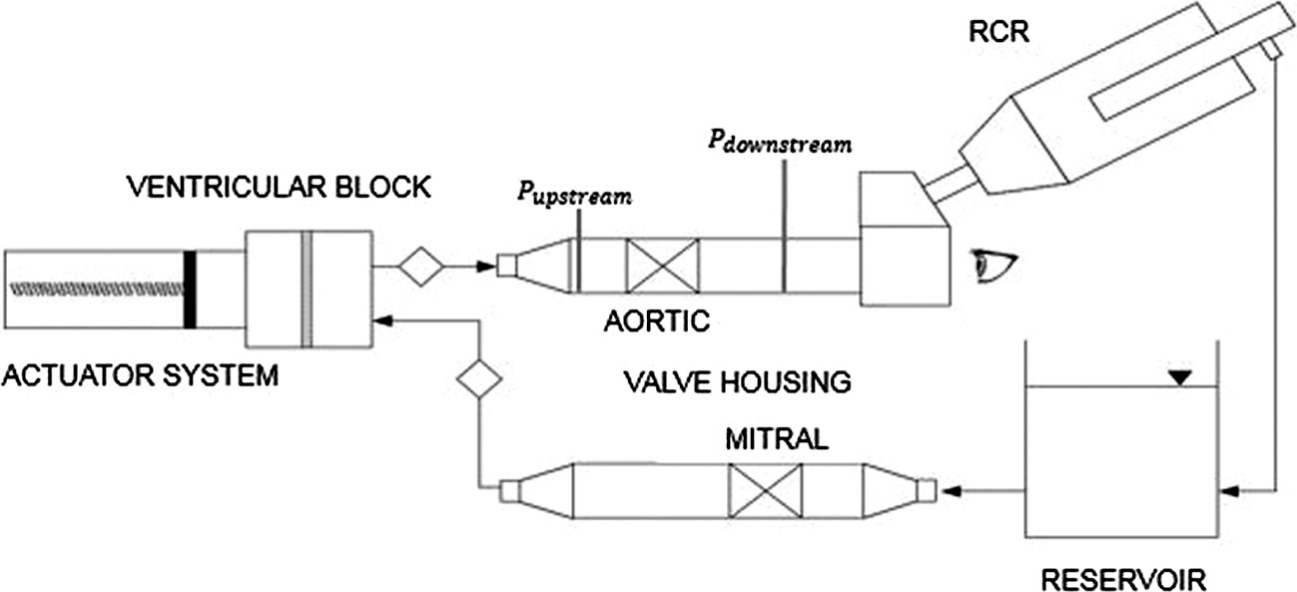
Sketch of the test bench for the pulsatile flow experiment; the locations of upstream and downstream pressure probes are shown; the observation point from which pictures were taken is indicated by the eye symbol.

Pulsatile tests were conducted on an in-house pulse duplicator (De Gaetano et al., 2015b). The following components were part of the pulse duplicator (Fig. 2): (i) a driving system made of a piston pump; (ii) a ventricular element, simulating the left ventricle; (iii) an aortic valve housing; (iv) a Resistance-Compliance-Resistance (RCR) analogue to replicate the aortic resistance, the compliance of the cardiovascular system, and the peripheral resistances; (v) a reservoir simulating the left atrium and (vi) a mitral valve housing. A dedicated software allows the user to set different flow rate waveforms with different frequencies. Systolic and diastolic flow rates were replicated with sinusoidal waveform. The pumping system was filled up with distilled water at 22 °C according to ISO5840:2015 Standard. The transvalvular pressure drop was measured at a constant frequency of 70 bpm and two flow rates: (i) 4 l/min (Test A), and (ii) 4.5 l/min (Test B). A high-speed video system (Canon EOS 70D, Tokyo, Japan) mounted in front of the aortic valve housing allowed to capture the valve kinematics during the *in vitro* tests.

### 2.3 Numerical simulations

Structural FE and FSI simulations were performed. The FE and FSI models were created with Rhinoceros 5.0 (Robert McNeel & Associates, USA) and discretized with Hypermesh (Altair Engineering, Inc., USA) and ICEM CFD 15.0 (ANSYS, Inc., Canonsburg PA, USA). All simulations were performed on an Intel Xeon workstation with 8 processors at 2.4 GHz using the commercial finite element solver LS-DYNA 971 Release 7.0 (LSTC, Livermore CA, USA and ANSYS, Inc., Canonsburg PA, USA).

**Fig. 3:**
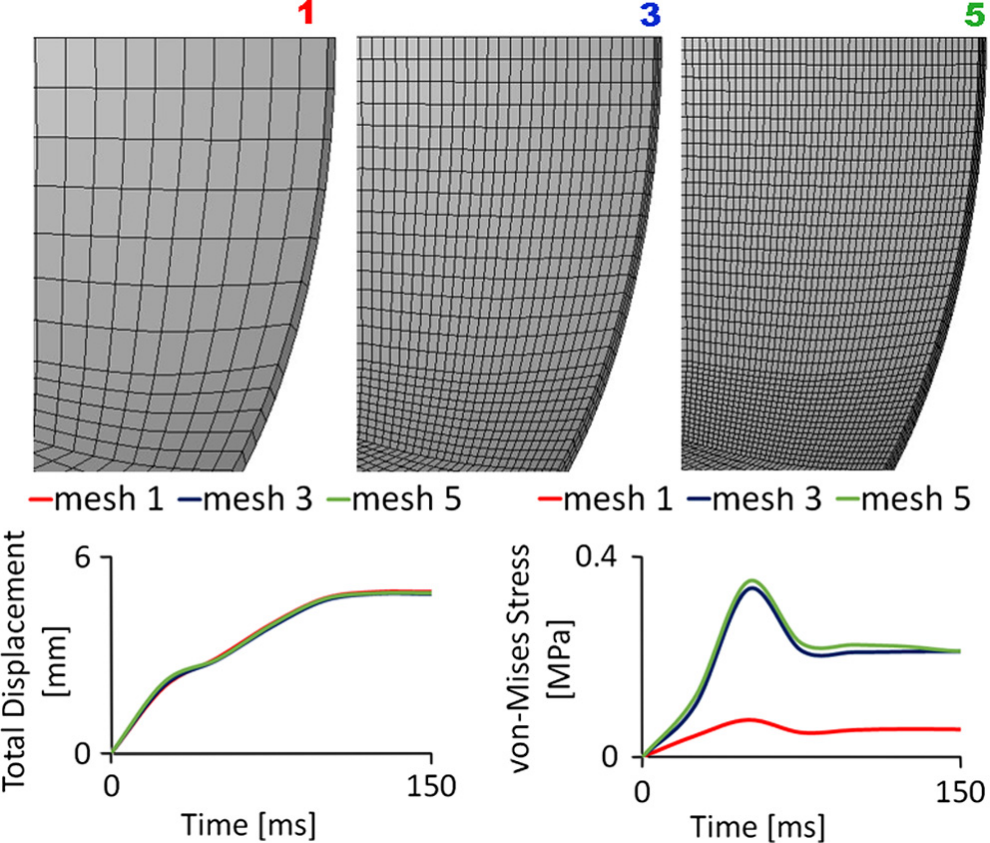
Results of the sensitivity analysis on the valve mesh. Mesh 1 has 12,420 elements and one element through thickness, Mesh 2 has 141,810 elements and three elements through thickness, and Mesh 5 has 600,456 elements and five elements through thickness. Details of the leaflets meshes are shown in the top panel. The total displacement (bottom left panel) and the von Mises stress (bottom right panel) at the middle of the free edge of the same leaflet for half cycle are depicted.

The valve and its housing were modelled using the actual dimensions of the samples (Table 1). The valve was modelled with 141,810 8-node hexahedral solid elements with both reduced and fully integrated (Fig.1b) to prevent hourglass problems. A mesh sensitivity analysis was performed for the valve on three different models with coarse (12,420 elements with one element in the leaflet thickness – Mesh 1 in Fig. 3), medium (141,810 elements with three elements in the leaflet thickness – Mesh 3 in Fig. 3) and fine (600,456 elements with five elements in the leaflet thickness of – Mesh 5 in Fig. 3) meshes. Results showed the independency of the mesh for the displacement of the leaflets, and the medium mesh is enough to get reasonable results on stress situation of the valve.

**Table 1:** Dimensions of the PHV.

PHV profiles	Value (mm)
Total height	14
Internal diameter (tissue annulus diameter)	23
External diameter	32
Thickness of the three leaflets	0.36–0.36–0.39
Height of leaflets	12

The PHV was considered as a linear elastic material with a Young modulus of 3.2 MPa obtained by fitting experimental data from Serrani et al. (2016), a density of 830 kg/m^3^, and a Poisson’s ratio of 0.49.

The valve housing, representing the aortic root, was considered as rigid, mimicking the experimental setup, and was discretized with 26,352 quadrangular shell elements (Fig.1c).

**Fig. 4:**
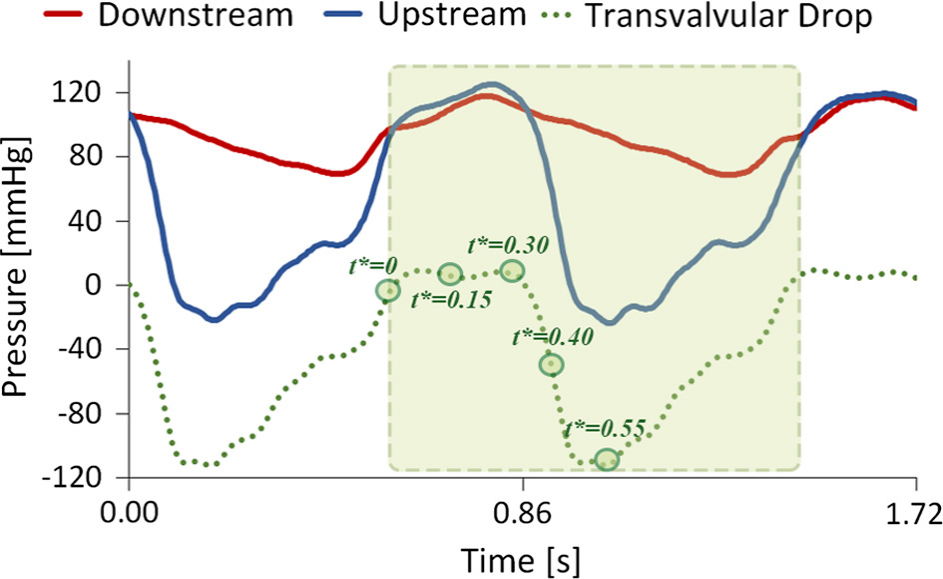
Experimental pressure curves imposed as boundary conditions at the inlet and outlet sections of the fluid domain. Circles on the curve are the time points where results are analysed (0, 0.15, 0.30, 0.40, 0.55 s of a cardiac cycle) (green area). (For interpretation of the references to colour in this figure legend, the reader is referred to the web version of this article.)

For the FE simulations, the experimental transvalvular pressure drop (Fig. 4) was directly applied on the surfaces of the leaflets. Furthermore, the external ring of the valve (Fig.1a) was constrained in all directions, to mimic the fixing of the valve in the housing. A surface to surface self-contact between the leaflets was defined to simulate the valve closure.

A fluid domain containing the structural elements, i.e., the valve, was created (see Fig.1d). It consisted of a control volume with an inlet and an outlet part at its ends. The independence of the fluid-dynamic results from the element size of the fluid mesh was performed using the optimal structural mesh determined previously. The sensitivity analysis of the fluid mesh indicated that a control volume discretized with 110,304 8-node hexahedral Eulerian elements with single integration point and 0.37 mm as minimum characteristic length was sufficient. The properties of water (density of 1000 kg/m^3^ and dynamic viscosity of 0.001 Pa s) were assigned to the fluid, modelled as a Newtonian fluid; a bulk modulus of 22 MPa, instead of 22 GPa, was used to reduce the simulation time, as proposed by Lau et al. (2010). Two complete cycles were simulated imposing the experimental pressure waveforms on the fluid inlet and outlet sections (Fig. 4). A no-slip boundary condition was applied on the fluid nodes located at the external surface of the aortic root (the valve housing). Two penalty couplings were applied to set the interaction between the valve and the fluid and between the compartment and the fluid. FSI simulations were performed using the immerse finite element method implemented within LS-DYNA.

The two test conditions (A and B), were reproduced with both FE and FSI simulations. Two cycles of 0.857 s each according to the frequency of the *in vitro* test ensured a stable response of the simulations. As for the results, the valve kinematics, the stress distribution, and the geometric orifice area (GOA) (Garcia and Kadem, 2006) were chosen to compare the *in vitro* and *in silico* tests at different time points as shown in Fig. 4.

## 3 Results

### 3.1 Valve kinematics

**Fig. 5:**
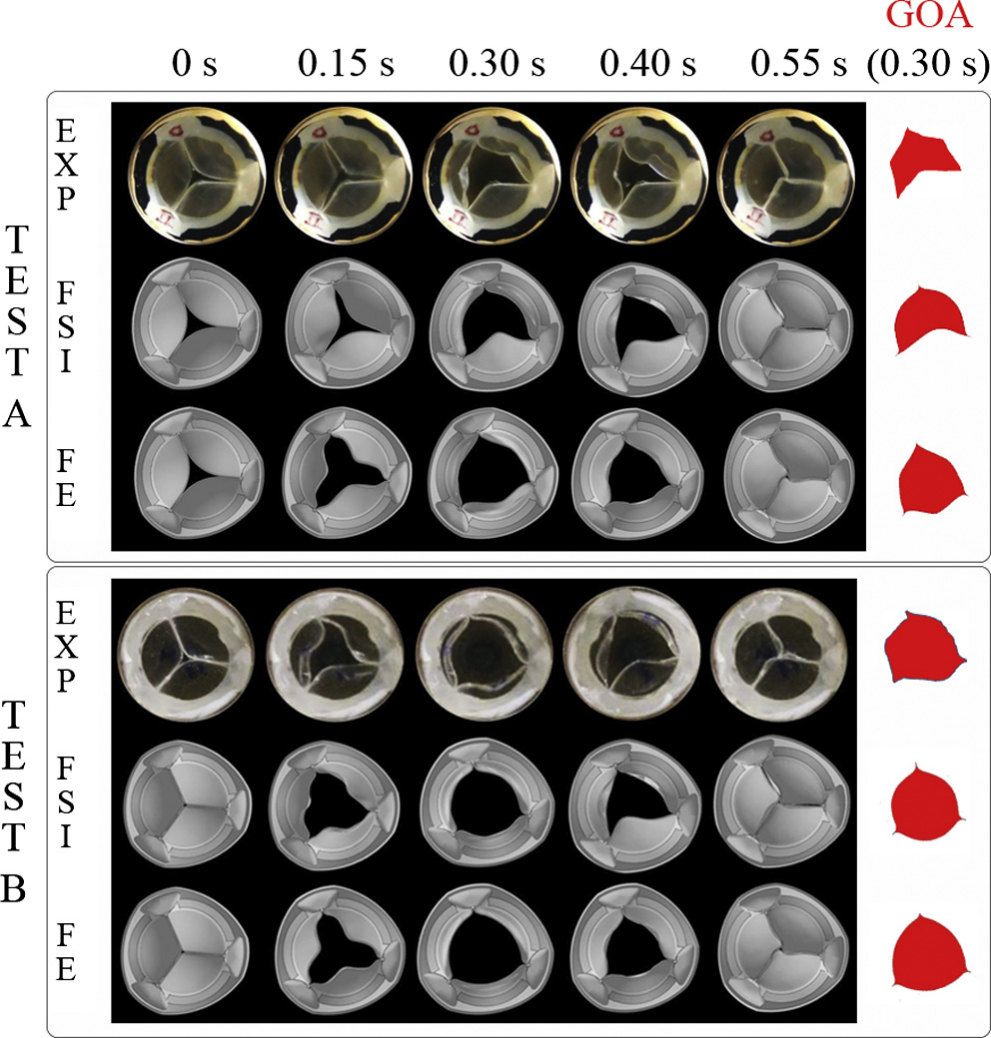
PHV kinematics for the *in vitro* tests (EXP), FSI and FE analyses at five time points for both test cases (Test A with 4 l/min and Test B with 4.5 l/min). Geometric Orifice Areas (GOA) at the maximum opening are also shown.

For test A, only two out of the three leaflets were fully opened (Fig. 5 Test A – EXP time 0.30 s), while for test B the three leaflets were fully and symmetrically opened (Fig. 5 Test B – EXP time 0.30 s).

Comparison with numerical results showed better similarity for the FSI than for the FE simulation (Fig. 5). For test A, only the FSI simulation reproduced the asymmetric opening observed in the experiment, with the thicker leaflet remaining closed (Fig. 5 Test A - FSI). For the FE analysis, the pressure imposed on the leaflets opened the valve fully (Fig. 5 Test A - FE). On the contrary, for test B, both the FSI and the FE analysis (Fig. 5 Test B – FSI and FE) reproduced the total opening of the vale observed in the *in vitro* experiment, but only the FSI simulation was able to reproduce the asymmetric opening and closing of the valve.

### 3.2 Evaluation of geometric orifice area

The GOA was evaluated by analysing the images from the *in vitro* tests and projecting the free-margin of the three leaflets on a plane parallel to the annulus. The same procedure was applied to the images from the numerical simulations. For test A, the difference between FSI and experimental results at the maximum opening area (time = 0.30 s) was only a 5% as oppose to a 46.25% for the FE analysis (Table 2). For test B, differences in the GOA between experiments and simulations were found to be 1.25% and 8.75% for the FSI and the FE analysis, respectively (Table 2). Furthermore, the shape of the reconstructed opening area from the FSI simulations (Fig. 5 GOA) resembled the experimental one closely. In Table 2 the GOA calculated at additional time points during opening and closing (0.15 s and 0.40) are reported. Results from Table 2 confirm the trend described previously.

**Table 2:** Geometric Orifice Areas (GEO) and Maximum Principal Stress for the two test cases (Test A with 4 l/min and Test B with 4.5 l/min).

Model	GOA (cm^2^) time = 0.15 s	GOA (cm^2^) time = 0.30 s	GOA (cm^2^) time = 0.40 s	Von-Mises Stress (MPa) time = 0.55 s
*TEST A*				
EXP	0.45	1.60	1.72	–
FSI	0.47	1.68	1.81	0.8510
	*Error* = *4.44%*	*Error* = *5%*	*Error* = *5.23%*	
STR	1.07	2.34	1.81	0.7130
	*Error* = *137.77%*	*Error* = *46.25%*	*Error* = *5.23%*	

*TEST B*				
EXP	1.70	2.40	1.79	–
FSI	1.84	2.43	1.81	0.8557
	*Error* = *8.23%*	*Error* = *1.25%*	*Error* = *1.12%*	
STR	1.86	2.61	1.82	0.7882
	*Error* = *9.41%*	*Error* = *8.75%*	*Error* = *1.68%*	

### 3.3 Stress fields on the valve

**Fig. 6:**
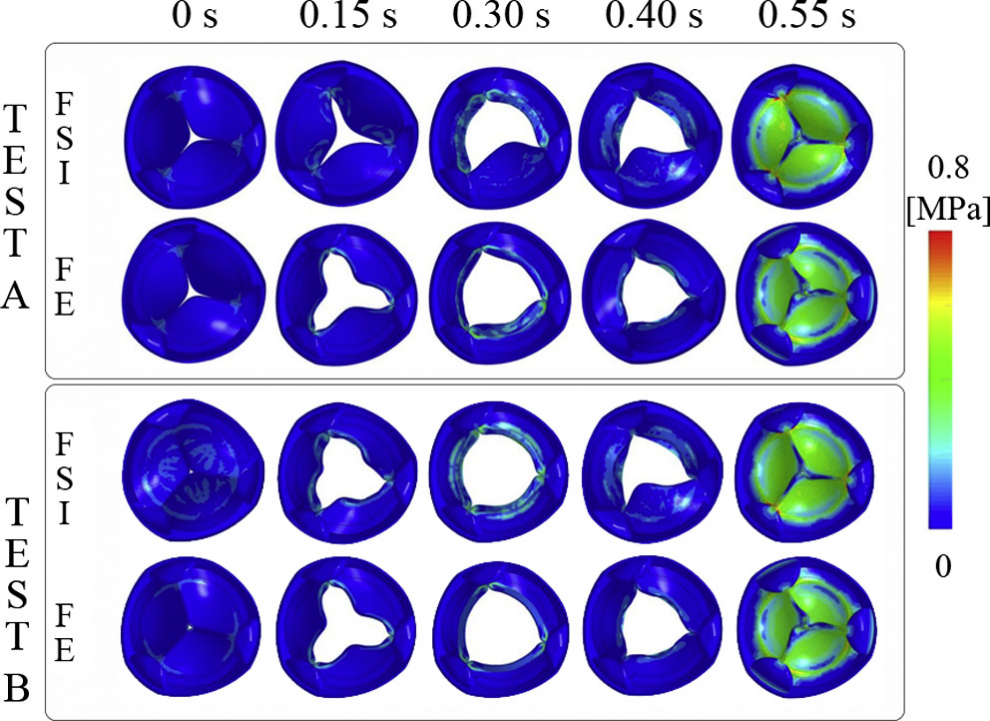
Von-Mises Stress distribution on the valve in the FSI and FE analyses for Test A and B.

**Fig. 7:**
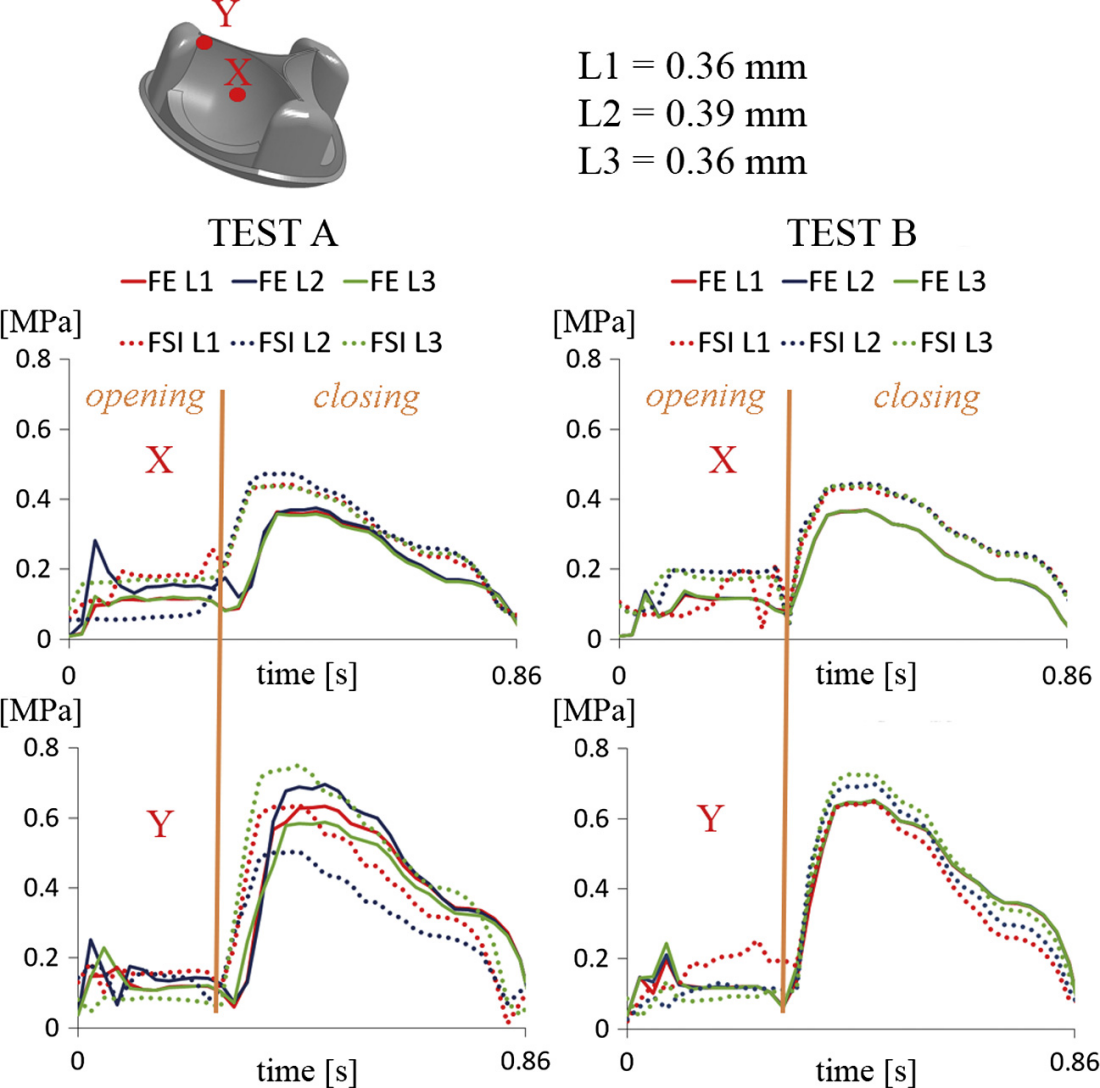
Von Mises Stress distribution on each leaflet mid-point (X) and on each leaflet point near the pillar (Y) for the FE and FSI simulation for Test A (left) and Test B (right).

The Von-Mises Stress field for all numerical simulations are shown in Fig. 6. A similar distribution was found for both the FSI and the FE analyses. However, the peak stress area, which was always located near the three pillars in the valve during its maximum closing, was slightly higher for the FSI models (0.851 MPa Test A and 0.856 MPa Test B) than for the structural ones (0.713 MPa Test A and 0.788 MPa Test B). For a more quantitative analysis, traces of the Von-Mises stress for elements located at the middle and near the pillars of each leaflet (point X and Y in Fig. 7) have been obtained. The comparison of these traces show the same general trend for both simulations for both test cases. However, the stress distribution clearly reflects the difference in valve kinematics during the opening phase between FE and FSI simulations (Fig. 7). That is, the symmetry of the three leaflets in the FE analyses and, on the contrary, the irregular leaflets behaviour in the FSI simulations. In addition, FSI predicts larger stress than FE simulations, in general.

## 4 Discussion

The present study demonstrates that FSI simulations are more appropriate than FE analyses to describe the real behaviour of a PHVs. Although in recent years numerical structural studies concerning the heart valve have been proposed (Gunning et al., 2014, Wang et al., 2015, Morganti et al., 2014), they cannot fully reproduce a realistic loading on the valve since these analyses neglect the influence of the fluid and its interaction with the valve. A hydrodynamic pulsatile test on a PHV was replicated with both FE and FSI simulations. Two tests (A and B) with different imposed flow rates were carried out in order to quantify the behaviour of valve opening for different loading conditions. The small difference between the two flow rates caused significant changes in the kinematic behaviour of the valve; results from *in vitro* tests indicate the existence of a flow rate threshold, between 4 and 4.5 l/min, for which the valve kinematics changes. Comparison was performed in terms of valve kinematics and stress distributions. Results have clearly shown that FSI simulations are closer to reality. As in previous works (Sturla et al., 2013, Lau et al., 2010), the GOA from FSI simulations resulted smaller than the one computed from FE analysis, being the difference more noticeable for small flow rates (Test A). These differences in GOA between FSI and FE simulations are primarily because the leaflets in the FE simulation are loaded with a homogenous pressure directly applied on them; whereas in the FSI, the leaflets support a non-homogenous pressure distribution as a result of the boundary conditions applied on the fluid domain. This spatial heterogeneity in the pressure caused by the interaction between the leaflets and the fluid causes the macroscopic differences observed on the valve kinematics. For larger flow rates the differences in GOA between FSI and FE reduce to less than 7% with GOA from FSI being smaller. It can be argued that the non-symmetric valve opening observed for the low flow rate is associated with differences in leaflet thickness present in the analysed model. Hence, if this thickness difference can be considered as non-physiological, then in the physiological scenario, the differences between FE and FSI may result irrelevant. To demonstrate the veracity of this point we run simulations with identical leaflets resulting also in a non-symmetrical opening of the valve for the FSI simulation (results not shown). This indicates that non-symmetric opening of the valve for flow rates below a threshold maybe associated with an intrinsic instability of the leaflet structure when subjected to hydrodynamic loads. This behaviour has been reported in a number of experimental studies reported in literature (Yousefi et al., 2016b, Yousefi et al., 2016a, De Gaetano et al., 2015b, Jahren et al., 2016, Schäfer et al., 2017, Kuetting et al., 2014).

Numerical simulations provide additional information with respect to experiments, as for instance, the stress distribution in the PHV, or the areas of stress concentration. In this regard, higher values of stress were found in the FSI simulations with respect to those obtained in the FE simulation, in general. This observation correlates with a previous work by Lau et al. (2010). The other relevant differences in term of stress distribution are strictly correlated with the different kinematics of the valves. This kind of investigation will allow us to identify, for example, the most critical areas for a fatigue analysis of this type of devices.

**Fig. 8:**
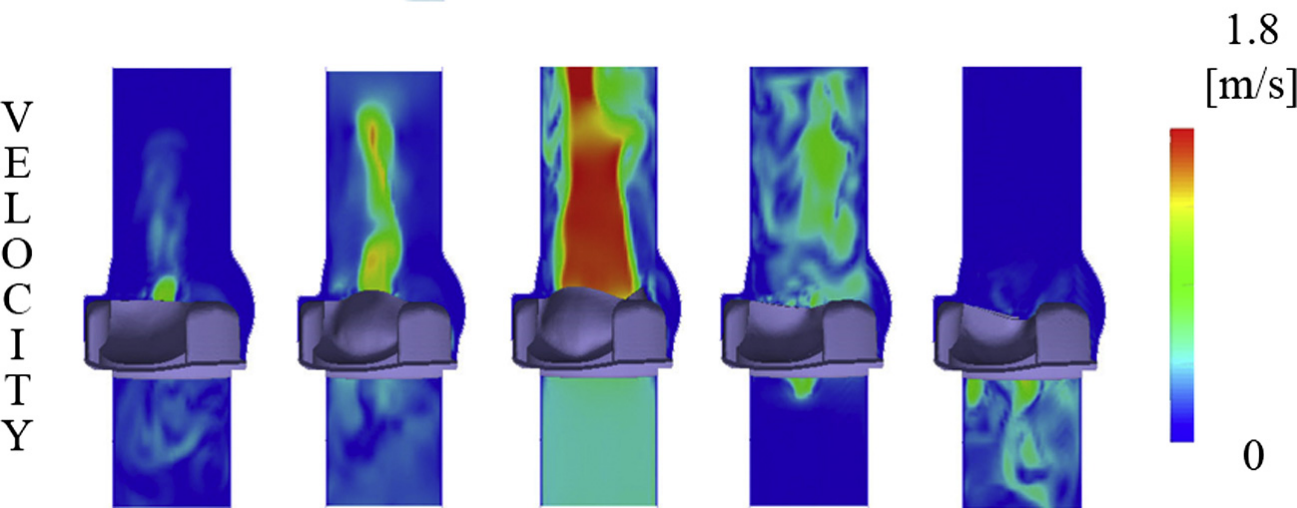
Velocity contour maps of the FSI simulations - Test B case.

Regarding the fluid domain, the purpose of this paper was not to study in detail the local fluid dynamics generated by the valve, but to show how FSI simulations can replicate the valve-fluid interaction while providing reasonable results (Fig. 8). However, the promising results obtained on the fluid domain stimulate further studies to investigate fluid dynamic aspects in more detail, as for instance valve regurgitation (bottom right panel in Fig. 8), vortex generation and the presence of recirculation areas. In addition, the determination of shear stresses is very important as a risk factor for hemolysis (Laflamme et al., 2015).

The study is not exempt from limitations. First, the polymeric valve was modelled as a homogeneous, linear elastic and isotropic material. The simplification of the polymer as a linear elastic model was used to stabilize the simulation due to the complexity of the contact among the leaflets as in Wu et al. (2016)). In this regard, specific anisotropic mechanical behaviour models will be adopted in the future by implementing a new constitutive law including the optimisation of the fibre direction in the polymer (Serrani et al., 2016). This solution represents a more elegant alternative than using a multilayer composite approach as in Wenk et al. (2012) since it provides continuity of the strain and stress fields. However, although we can expect differences in the stress values, the comparison between FE and FSI simulations is expected to remain the same. Secondly, a further process of validation for the fluid dynamics aspects (wall shear stress, velocity vectors) would be required with techniques as the particle image velocimetry (PIV) to compare experimental and numerical fluid dynamic results in a thorough way.

In conclusion, the relevance of performing validated FSI simulations relies on the possibility to investigate scenarios closer to the reality. In this regard, this work shows that GOA computed from FSI simulation is more accurate than that derived from a FE simulation. Furthermore, the importance of this work rests on the qualitative and quantitative validation of the *in silico* models using experimental data. Indeed, *in silico* studies allow to analyse specific aspects from both the structural and the fluid-dynamic point of view which cannot always be investigated by *in vitro* tests, as for instance the stress distribution on the valve or the shear stress in the fluid in the proximity of the leaflets. Lastly, FSI simulations may become particularly useful during the different steps of prosthetic heart valve design, as well as to refine and propose new standards for the preclinical evaluation of medical devices.

## Conflict of interest

None.
